# Prosodic Phrasing of Good Speakers in English and Czech

**DOI:** 10.3389/fpsyg.2022.857647

**Published:** 2022-03-24

**Authors:** Radek Skarnitzl, Hana Hledíková

**Affiliations:** ^1^Institute of Phonetics, Faculty of Arts, Charles University, Prague, Czechia; ^2^Department of English Language and ELT Methodology, Faculty of Arts, Charles University, Prague, Czechia

**Keywords:** prosodic phrasing, pitch range, melodic variability, speaking rate, Czech, English

## Abstract

Prosodic patterning is known to affect the impression that speakers make on their listeners. This study explores prosodic phrasing in good public speakers of American English and Czech. Czech is a West Slavic language whose intonation is reported to be flatter and prosodic phrases longer than in English. We analyzed prosodic characteristics of 10 speakers of Czech and American English who appeared in TED Talks, assuming such appearance to be a mark of a “good speaker.” Our objective was to see whether prosodic phrasing will be more similar in these public speeches between the two languages. We measured the length of prosodic phrases, speech rate in each phrase, and pitch range and melodic variability in the entire phrase, as well as in its pre-nuclear and nuclear portion. The number of syllables per phrase was higher in Czech than in English, although phrases were generally very short in both languages. The melodic indicators confirm smaller melodic variability in Czech even in publicly performed TED Talks. Overall, our results show that there are differences between Czech and English prosodic phrasing in good public speakers but that the genre also affects phrasing. Prosodic rendition—especially prosodic phrase length and melodic variability—is therefore a vital, albeit somewhat language-specific aspect of speech performance which public speakers should pay close attention to, both in their native language and in foreign languages.

## Introduction

The study of personal charisma has a long history. Originally, charisma was regarded as a supernatural quality, an in-born talent granted only to few (Weber, 1947). While this idea was soon abandoned and we know today that charisma is a skill which can be acquired and trained (e.g., Towler, 2003; Antonakis et al., 2011; Niebuhr et al., 2019; Niebuhr and Neitsch, 2020), a number of myths surrounding the nature of charisma persist (Michalsky and Niebuhr, 2019). It has been established that charisma has a positive impact on, among others, the electoral success of politicians (Sheafer, 2008), the willingness of external stakeholders to participate in a company (Fanelli and Misangyi, 2006), university students’ motivation and perception of their learning (Bolkan and Goodboy, 2014), the credibility of an advertised product (Gélinas-Chebat et al., 1996) or, sadly but not surprisingly, the attractiveness of radical preachers for their followers (Gendron, 2017).

It is an undeniable fact that charisma is closely related to communication skills. Several studies have compared the relative effect of content and delivery on the perception of charisma. In one of the first experimental studies, Holladay and Coombs (1994) combined visionary vs. non-visionary content with strong vs. weak delivery and found that strong delivery outweighed the effect of non-visionary content; in other words, delivery contributed more to the perception of charisma than content. Similar results were reported by Awamleh and Gardner (1999), who added organizational performance as a third factor determining a business leader’s charisma. Amon (2016) cited in Michalsky and Niebuhr (2019, p. 36) goes so far as to claim that “[t]he moment you open your mouth, all the visible elements become mere decoration.” In a more recent study concerning content and delivery, Caspi et al. (2019) extended the findings by offering a dual-process perspective: delivery is a stronger determiner of charisma because it is processed faster, more automatically than content whose processing requires conscious deliberation. According to the authors, the immediate impression based on the speaker’s delivery “anchors” the perception of the speaker’s charisma, which may only be modified if delivery and content are markedly misaligned.

In the above-mentioned studies, the components of delivery—apart from factors related directly to speech—included the maintenance of eye contact, gesticulation, and facial expressiveness, as well as relaxation and confidence of the speaker. While all these are key determinants of charismatic delivery, we will continue to focus only on speech characteristics. It is worth pointing out here, however, that the concept of charismatic speaker and charismatic speech is, to a certain extent, culture- or language-specific (Biadsy et al., 2008; D’Errico et al., 2013), and reported results should not be regarded as applicable universally.

For a long time, the advice featured in various manuals on rhetoric relied on impressionistic terms rather than on phonetic research; as shown by Niebuhr et al. (2017), however, respondents imagine very diverse concepts when describing the ideal voice as “rich,” “durable,” or “flexible.” It is only with advancing phonetic research of the last approximately two decades that the label “charismatic voice” has been acquiring more specific meaning, and rhetoric manuals have been able to provide more explicit and targeted advice (see Gilner, 2014; Lucas, 2015: Ch. 13; Lower Mekong Initiative, 2017: Unit 7).

Charismatic speech has been researched in relation to both segmental and prosodic aspects, with a rather strong bias for the latter. In a segmentally oriented study, Niebuhr (2017) found that a moderate degree of reductions in speech resulted in speakers being perceived as more sociable, composed and sincere (traits which correlate with speaker charisma) than in speech which featured no reductions, as well as strong reductions. Interestingly, this result thus lends only limited support to the popular adage “Speak clearly!” On the other hand, two studies comparing Steve Jobs (famous for his strong charisma) and Mark Zuckerberg (known rather for the opposite) found clearer articulation of stop consonants (Niebuhr et al., 2018b) and vowels (Niebuhr and Gonzalez, 2019) in the late Apple CEO, while Zuckerberg’s speech was characterized by less clear realizations of consonants and by a smaller vocalic space, respectively.

The above-mentioned exceptions notwithstanding, most research on charismatic speech has focused on prosodic characteristics or, as these are frequently called in rhetoric manuals, the tone of voice. Indeed, it is not surprising that good prosody has been recognized as contributing to high-quality speech the most; we may observe a parallel with second language pronunciation, where prosody has also become regarded as most beneficial in achieving learners’ intelligibility (Derwing and Rossiter, 2003). As prosody in good speakers is the topic of the present study, we will examine the relation between charismatic speech and prosody in more detail in the next section.

### Charismatic Speech and Prosody

Prosodic features of speech include in the perceptual domain, melodic organization, temporal characteristics (e.g., speech rate, rhythm, or the length of prosodic constituents), loudness, and voice quality, and their respective correlates in the acoustic domain, fundamental frequency (*f*_0_), duration, intensity, and spectral characteristics. It is especially features of the first two groups—melodic and temporal aspects of speech—that have been shown to be crucial in the process of communication, playing an important role in the impression the speaker makes on listeners.

Most of the prosodic characteristics of a good speaker are intuitive; in other words, it is not surprising which kinds of prosodic behavior boost a speaker’s charisma and which are detrimental to it. However, this does not apply to one’s pitch level in a straightforward way—there seems to be no *a priori* reason why either a lower or higher pitch level should positively affect a speaker’s charisma. Many rhetorical manuals advise their readers to lower the pitch of their voice: Barker (2011, p. 14) associates lower pitch with easier rapport creating and higher pitch with stress and tenseness in the voice; even the most current edition (Barker, 2019) advocates lower pitch repeatedly. Despite this, the majority of empirical research reports a positive correlation between speaking fundamental frequency and a speaker’s charisma. Higher pitch level is therefore associated with higher and not lower charisma ratings. This was found in numerous studies which focused on charisma in politicians, for example, in Jacques Chirac’s speeches (Touati, 1993), the debates of members of the Swedish parliament (Strangert and Gustafson, 2008), in the speech of nine candidates for the Democratic Party’s 2004 presidential nomination (Rosenberg and Hirschberg, 2009), in a comparison of a French and Italian politician (D’Errico et al., 2013). A positive relationship between higher speaking fundamental frequency and perceived charisma was extended to business contexts: in a detailed analysis of Steve Jobs’ voice, Niebuhr et al. (2016) found a markedly higher pitch level in Steve Jobs than in the reference speakers. It must be emphasized, however, that the higher pitch level does not consist in a mere upscaling of *f*_0_ values: as found by Mixdorff et al. (2018, p. 817) in another comparison of Jobs and Zuckerberg, the higher *f*_0_ mean may consist in the lowering of the *f*_0_ baseline “and modifying the *f*_0_ slopes of pitch accents and initial and final boundary tones such that they get longer, higher, and arrive faster at a high *f*_0_ level” (*ibid*).

The latter finding brings us to *f*_0_ range, a crucial factor in the creation of a speaker’s charisma. According to all of the studies cited in the previous paragraph and others (e.g., Biadsy et al., 2008; Niebuhr et al., 2018a), higher charisma is associated with a larger melodic range. Given the nature of the speakers reported in this paper, it is worth mentioning a study by Berger et al. (2019), in which prosodic characteristics of the speech of YouTube video creators were correlated with the number of subscribers of their channels and the number of views and likes received. A significant positive correlation with *f*_0_ range was only found with the first indicator, subscriber count. It is also important to draw attention to what Niebuhr et al. (2019) call overdose thresholds: there are limits to the positive correlations. One may imagine that an excessively large pitch range will make the speaker sound affected and untrustworthy; given the different habitual *f*_0_ range in different languages (Mennen et al., 2012; Andreeva et al., 2014; Volín et al., 2015), the threshold is also likely to be language- or culture-specific (Grabe et al., 2003; Chen et al., 2004).

Another aspect which is related to melodic patterning and which has been found to correlate with perceived speaker charisma is focused words, or emphatic accents. Specifically, it appears that a higher number of these emphatic accents, as well as higher melodic peaks in them yield higher charisma ratings (Strangert and Gustafson, 2008). In addition, Niebuhr et al. (2016) documented not only a high number of instances of emphatic accentuation in Steve Jobs’ speeches, but also all of its subtypes, with a prevalence of trust-seeking reinforcement and positive intensification.

Concerning the temporal organization of charismatic speech, faster speech rate appears to be preferred by listeners (Biadsy et al., 2008; Rosenberg and Hirschberg, 2009; Niebuhr et al., 2016), although it is obvious that an overdose threshold will apply in speech rate as well. In Steve Jobs’ speech, Mixdorff et al. (2018) documented a strong and symmetric acceleration and deceleration in speech rate and a higher variability in speech rate, as compared with Mark Zuckerberg’s speech.

Another key descriptor which is associated with good speakers is fluency, which may be defined as a natural flow of speech. According to De Jong and Mora (2019, p. 228), spoken fluency largely depends “on speakers’ ability to execute the conceptualization and formulation of messages effectively and on their ability to translate formulated messages into articulatory actions smoothly during the speech production process.” A number of fluency measures have been proposed, particularly in the domain of second language performance (Suzuki et al., 2021); some of these include articulation rate and speech rate, mean length of run between pauses, or the phonation time ratio, as well as the frequency of various disfluency phenomena such as hesitations, repetitions or repairs, prolongations, or false starts (McDougall and Duckworth, 2017). It is not surprising that more charismatic speakers produce fewer of such disfluencies, as shown for instance by Strangert and Gustafson (2008).

In their summary of good speaker characteristics, Niebuhr et al. (2017, p. 10) mention, with respect to fluency, that “inferred from the evidence we already have, the rhetorical advice to ‘speak fluently’ can be translated into ‘split up your sentences into short phrases of no more than 4–5 words (about 2.0–2.5 s)’.” Similarly, Niebuhr et al. (2019, p. 2) state in their summary of research that “prosodic-phrase durations… are negatively correlated with perceived speaker charisma.” It is interesting, however, that there seems to be very little empirical evidence to support this claim. Some exceptions are the study by Strangert (2005), whose comparison of two skilled speakers revealed rather short phrases governed by the semantic rather than syntactic structure, as well as the comparison of Jobs and Zuckerberg by Niebuhr et al. (2016). Contrariwise, however, Rosenberg and Hirschberg (2009) report that the number of words per phrase was significantly and positively correlated with ratings of charisma.

It remains to be pointed out that there may be different types of charisma. For example, D’Errico et al. (2013) compared the speech of the American politicians Barack Obama and Donald Trump, as well as that of the Italian politicians Paolo Gentiloni and Matteo Salvini. While all of them may be regarded as charismatic in some sense, Obama’s and Gentiloni’s humility stands in stark contrast to Trump’s and Salvini’s dominance. The authors found marked differences between the humble and dominant politicians: the former used shorter utterances, more disfluencies, fewer stressed words, and speech which was less loud and slower than the latter.

To summarize the main findings of prosodic research into good speakers, “variation and variety are key concepts in creating a charismatic impact” (Niebuhr et al., 2016, p. 376). The validity of this adage is also supported by speech synthesis and resynthesis experiments: speech generated in a controlled way, using a large pitch range and few disfluencies, was rated more positively on charisma-related traits (Strangert and Gustafson, 2008; Fischer et al., 2019).

### Prosodic Phrase as a Central Unit of Prosody

Prosodic features serve the role of organizing the flow of speech and giving it structure by dividing it into smaller units, which are called prosodic phrases, prosodic units, tone units, thought groups etc. The prosodic phrase is defined as “the domain of a perceptually coherent intonational contour” (Shattuck-Hufnagel and Turk, 1996, p. 210) and is delimited by prosodic boundaries. The strongest prominence of the prosodic phrase, realized on the tonic syllable, is called the nuclear stress. Nuclear stress tends to occur on the stressed syllable of the last content word in a phrase (Féry, 2017, p. 61) but may also be placed on other words to give it emphasis. Prosodic phrases are separated by prosodic boundaries, which are usually signaled by melodic and temporal features (melodic movements and final deceleration or lengthening, respectively), sometimes by a pause.

From the perspective of speech production, empirical evidence suggests that when speakers plan the lexical content of a stretch of speech, constructions which correspond in their size to a clause, with about four to six words, are preferred (Nooteboom, 1995; Pawley and Syder, 2000); it is not unreasonable to draw a parallel between a clause and prosodic phrase here. With this one clause at a time hypothesis, Pawley and Syder point to the crucial role of a fluent unit which people can handle in a single focus of consciousness. In other words, prosodic phrasing tends to reflect the syntactic or information structure of a sentence (see also Cresti, 2018), but the authors claim that it is primarily not grammatical structure but a processing limit on our language planning that yields the typical prosodic structure of speech. We should point out that prosodic planning also depends on the capacity of speakers’ working memory (Swets et al., 2007; Petrone et al., 2011).

From the perspective of speech perception, prosodic phrasing has been shown to play a significant role in listeners’ comprehension. Numerous experiments which have been conducted since the 1960s and 70s showed that speech is processed faster and its contents are recalled with higher success rate if it is presented with clear phrasal prosody (e.g., O’Connell et al., 1968; Zurif and Mendelsohn, 1972; Leonard, 1974; Sturges and Martin, 1974; Reeves et al., 2000; Krivokapić, 2007). It therefore appears that it is phrasal prosody that provides the basic structure which allows us to hold a sequence of heard words in memory (Frazier et al., 2006).

Apart from being regulated by quasi-universal requirements of speech production and perception, prosodic phrasing differs to some extent between languages (Jun, 2003). Such differences may consist, for example, in the mapping between syntactic and prosodic structure, or in the effect of focus on the possibility of prosodic boundary placement. In this study, we analyze prosodic phrasing in “good speakers” of English and Czech; in the next section, we will therefore briefly compare the prosodic patterns of these two languages.

### Prosodic Patterns in English and Czech

If we were to express the difference between English and Czech prosodic characteristics using a single impressionistic word, we could refer to English as vivid and to Czech as monotonous. At the level of individual words, lexical stress is manifested in English by higher *f*_0_ level, longer duration, and shallower spectral slope (e.g., Eriksson and Heldner, 2015), while in Czech, the stressed syllable bears no prominence in any acoustic domain (Skarnitzl and Eriksson, 2017; Skarnitzl, 2018). This is not surprising given the fact that Czech is a language with stress fixed to the first syllable of a prosodic word and no contrastive function (*cf.* Cutler, 2005). Recent evidence also suggests that it may be suitable to talk about accentual groups rather than stress groups in Czech, with groups of words sometimes joined into one prosodic unit (Volín and Skarnitzl, 2020). In terms of rhythmic properties, English is a language characterized by large differences in syllable durations, with long stressed syllables and unstressed ones reduced in duration, as well as quality; traditionally English has been described as a stress-based language (Dauer, 1983). Czech has phonological vowel length, although phonologically long vowels are considerably less frequent in connected speech (see Volín, 2010, p. 45), and no systematic vowel reduction; traditionally Czech has been described as a syllable-based language, in spite of its syllabic complexity (Šturm and Lukeš, 2017; see also Dankovičová and Dellwo, 1999).

The monotonousness of Czech is evident not only in the weak to absent prominence contrasts, but particularly in the melodic domain. Compared to English, Czech intonation comes across as rather flat, and there seem to be two reasons for this. First, pitch range is significantly narrower in Czech: in a comparison of English and Czech professional newsreaders, Volín et al. (2015) report the 80-percentile range (i.e., the difference between the 90th and 10th percentile) to be 7.1 semitones (ST) for British females and 8.1 ST for British males, while the values in Czech were 5.2 ST and 6.1 ST, respectively. Melody seems to be even flatter in spontaneous speech: unpublished data show that in 56 out of 100 male speakers, 80-percentile range does not exceed 5 ST. The second reason for the monotonous impression is the fact that, apart from flatter melody, Czech is also characterized by longer prosodic phrases. In a comparison of British English and Czech radio newsreaders, Volín (2019) found that prosodic phrases in English were, on average, by nearly 40% longer in Czech than in English, with the mean lengths being 10.8 and 7.8 syllables, respectively; interestingly, there was no significant difference, however, when phrase length was expressed in words (4.6 in Czech and 4.5 in English). Volín also examined news reading by non-professional Czech speakers and reported even longer prosodic phrases, with the mean being 12.9 syllables and 5.4 words. It seems natural that segmenting utterances into a smaller number of longer prosodic phrases will further contribute to the perceived monotonousness of Czech intonation, as there are longer stretches of speech without salient melodic movements.

### Research Questions

It was mentioned in “Charismatic Speech and Prosody” that the association of good speaker qualities with short prosodic phrases is, to the best of our knowledge (and also as confirmed by Niebuhr, November 2021, personal communication), based on rather anecdotal evidence. At the same time, observations of everyday Czech speech indicate that prosodic phrases are rather long in Czech, considerably longer than in English. Combining these two gaps in our knowledge, the general objective of the present study is to compare prosodic phrasing in good speakers of English and Czech, in the genre of public speaking.

Specifically, then, we are asking whether more monotonous prosody is also observed in good speakers of Czech, as compared with speakers of English, or whether the communicative demands of public speaking lead to a different prosodic behavior than observed in ordinary Czech speech. In other words, our goal is to compare genre- and language-specific tendencies in skilled speakers of Czech and English. Genre-based differences would be in agreement with the results of De Pijper and Sanderman (1994), who showed that professional speakers produced more prosodic cues and more salient ones than non-professional speakers. On the other hand, language-based differences may persist, with Czech and English speakers in the same genre still manifesting different prosodic tendencies. We will use measures of prosodic phrase length, articulation rate, pitch range, and melodic variability in the domain of the prosodic phrase to describe good public speakers’ prosodic phrasing in the two languages.

One of the contributions of this study is extending the scope of melodic measures. Apart from applying a still relatively new measure of melodic variability (see “Analyses” below), we focus not only on entire prosodic phrases, but also separately on their nuclear part (i.e., in the syllables which carry the nuclear tone of each phrase) and their pre-nuclear field (i.e., in all the syllables preceding the nuclear syllable). It is especially the analysis in the pre-nuclear field that makes this study different from the results reported by Hledíková (2019), on which this study is based. The motivation for including the pre-nuclear field comes from the informal observation that the flatness of Czech intonation may not be captured by traditional indicators of pitch range like standard deviation: the flatness may be limited only to the pre-nuclear field, with little melodic movement apart from the nuclear tone itself.

## Materials and Methods

### Material

This study is based on TED Talks delivered in American English and Czech. In TED Talk events, speakers present a topic in an attractive and entertaining way to a general audience, in a relatively limited time span (typically between 15 and 20 min). In the first stage, we selected 15 speakers of each language; this selection was based on subjectively perceived speaker quality. We assumed that already the fact that a speaker was invited to a TED Talk event provides a certain guarantee of their speaking competence: in fact, TED Talks have been described in a recent study as “the pinnacle of public speaking” (Tsai, 2015).^1^ However, to further ensure the high quality of the speakers’ performance, we conducted an informal listening test with eight listeners per language. The respondents were played 30-s segments and asked to express their willingness to employ the speaker as their spokesperson on a 7-point scale. Ten TED Talk speakers of each language who received the highest mean score were chosen for subsequent analysis; this corresponded to six males and four females speakers in English, and nine males and one female speakers in Czech. Due to the gender imbalance, possible differences between female and male speakers will not be examined.

The recordings were divided into shorter segments of approximately 1 min and forced-aligned using P2FA for English (Yuan and Liberman, 2008) and Prague Labeller for Czech (Pollák et al., 2007). Five minutes of speech per speaker were selected; the first 2 min were not analyzed because the speaker may need time to “get started” and find his speaking style.

### Analyses

Prosodic boundaries were labeled manually in Praat (Boersma and Weenink, 2019), using break indices 3 and 4 in line with ToBI conventions for minor and major prosodic breaks, respectively; any prosodic discontinuities were marked with a “p” (Beckman and Elam, 1997, p. 32). The labeling was carried out by the second author, and any uncertainties were settled in a joint analysis of the two authors. We also marked the syllable carrying nuclear stress in each phrase. A Praat script was then used to extract the following measures from the annotated data:

- number of syllables per prosodic phrase;
- number of words per prosodic phrase;
- articulation rate in syllables/s;
- *f*_0_ standard deviation (SD) in each prosodic phrase in ST;
- Cumulative Slope Index (CSI) in each prosodic phrase in ST/syllable; CSI corresponds to the sum of absolute frequency differences between subsequent *f*_0_ points divided by the number of syllables and thus captures melodic variability better than the more traditional measures, as it takes into account multiple melodic movements in a phrase (Hruška and Bořil, 2017);
- *f*_0_ SD in the nuclear part of the phrase in ST; and
- *f*_0_ SD in the pre-nuclear part of the phrase in ST.

Fundamental frequency was extracted using default settings for autocorrelation in Praat, only with the ceiling lowered (320 Hz for male and 450 Hz for female speakers). The extracted Pitch objects were smoothed using a 10-Hz filter to eliminate microprosodic fluctuations in *f*_0_, interpolated, and converted into PitchTier objects which were used to measure the SD of *f*_0_ in ST.

Linear mixed-effects (LME) models were constructed for the statistical analyses, using (R Core Team, 2017) and the *lme4* package (Bates et al., 2015). The prosodic measures listed above served as dependent variables. There were two fixed effects, language (English, Czech) and prosodic break type (BI4, BI3). Random effects included speaker intercept (since speakers may differ in their prosodic behavior) and by-speaker slope for the effect of prosodic break type (since speakers may differ in their realization of each type of prosodic break). Residual plots were visually inspected for deviations from normality and homoscedasticity. The significance of individual effects or interactions was tested by comparing the full model to a reduced model with the given factor or interaction excluded. We conducted Tukey post-hoc tests using the *multcomp* package (Hothorn et al., 2008) to test specific pairwise comparisons. Plots showing mean values of the measured variables and their confidence intervals were created using the *effects* package (Fox, 2003) and visualized with *ggplot2* (Wickham, 2009).

## Results

Results will be presented in two main sections focusing gradually on temporal measures (length of prosodic phrases and articulation rate) and on melodic measures. They will always be displayed separately for major (BI4) and minor (BI3) prosodic breaks. In addition, a separate analysis will be presented for phrases which feature no disfluencies (in other words, we are also interested in the prosodic behavior of fully developed and realized phrases); since disfluencies occurred prevalently in minor phrases (i.e., those ending with a minor prosodic break), only phrases ending in BI4 without disfluencies (marked BI4-d below) will be considered in these partial analyses. “Individual Prosodic Profiles” will be dedicated to assessing prosodic phrase length and melodic range in a single comparison, so as to focus on possible individual differences between our TED Talk speakers.

### Temporal Aspects of Phrasing

**Figure 1:**
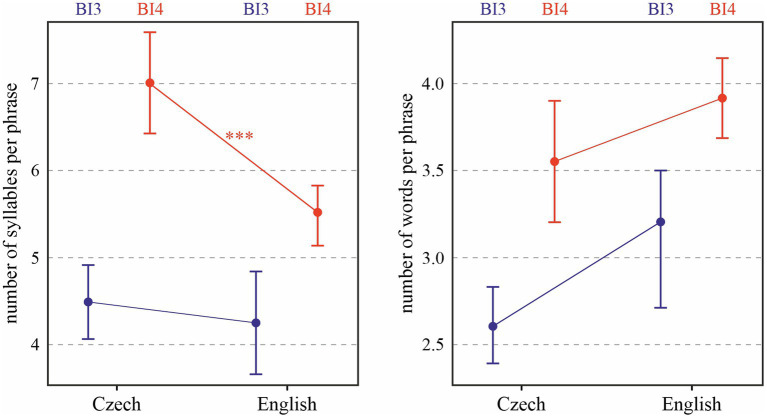
Number of syllables (left) and words (right) per prosodic phrase depending on Language and Prosodic break type (BI3 in blue; BI4 in red). ^***^p < 0.001.

Mean length of prosodic phrases in both languages, expressed in syllables and words, is shown in Figure 1. It is obvious that the syllable and word levels provide different results. As for syllables (shown on the left of the figure), both Language [*χ*^2^(1) = 7.80, *p* < 0.01] and prosodic break type [*χ*^2^(1) = 25.18, *p* < 0.0001] turned out to be significant predictors of phrase length. Phrases delivered by our English speakers are shorter, on average, by 0.89 (± 0.25 standard errors) syllables than those uttered by the Czech speakers, and phrases ending in a stronger prosodic break are longer by 0.5–3.7 syllables (with differences between individual speakers). The figure also suggests an interaction between language and prosodic break type, which is indeed significant: *χ*^2^(1) = 6.98, *p* < 0.01. Tukey post-hoc tests reveal that it is the BI4 context (i.e., major prosodic breaks) where Czech and English speakers’ phrases differ in length (*p* < 0.001).

When expressed in the number of words, the effect of Language on prosodic phrase length is marginally significant [*χ*^2^(1) = 2.88, *p* < 0.1], with phrases in English longer by 0.43 word on average (± 0.13 standard errors). This is not surprising, because although Czech phrases are longer when expressed in syllables, the analytical English uses many short words with a grammatical function, such as articles or prepositions, as opposed to the synthetic Czech which uses inflections to express grammatical relationships. The results are similar for the effect of prosodic break type [*χ*^2^(1) = 23.47, *p* < 0.0001], with phrases ending in BI4 being longer by 0.5–1.6 words than those ending in BI3. Tukey post-hoc tests reveal no significant effects of language (*p* > 0.1).

**Figure 2:**
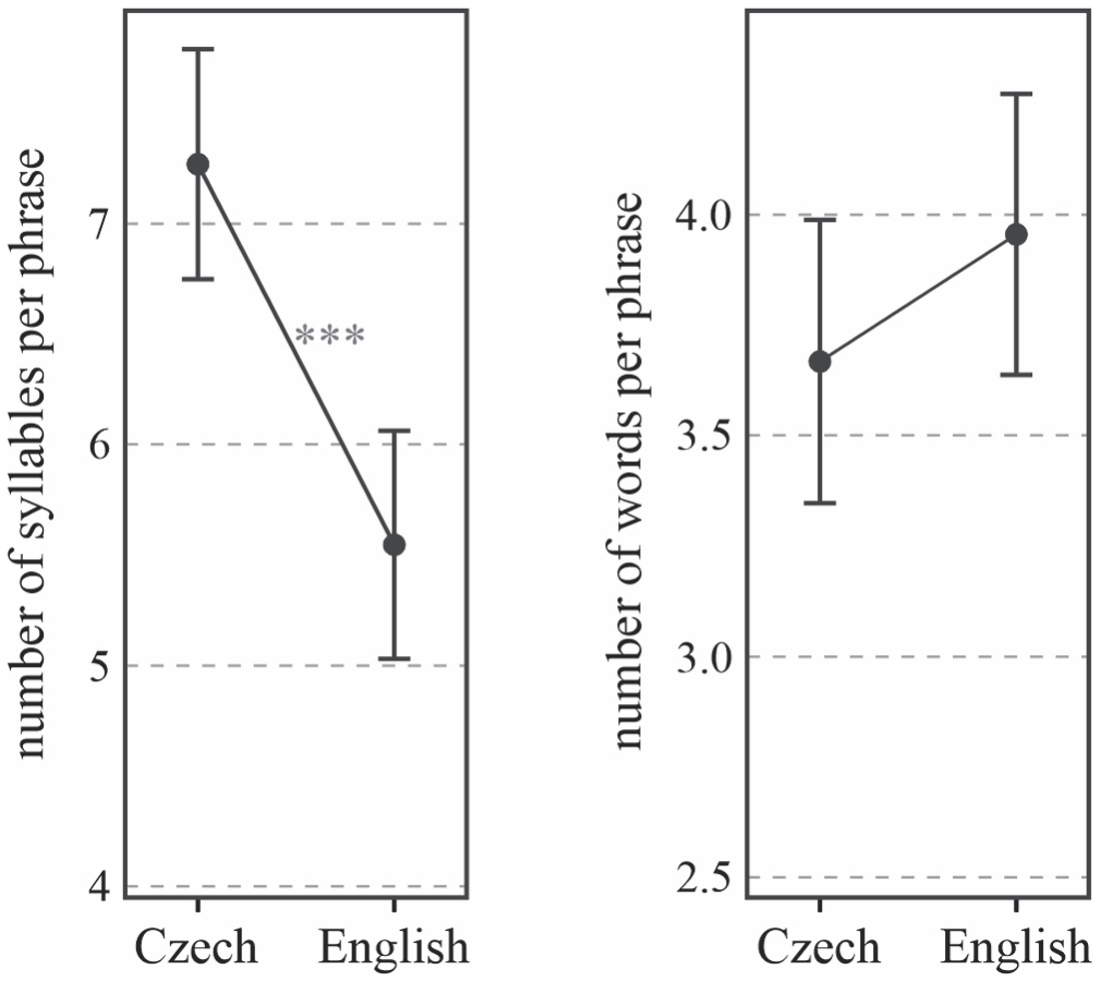
Number of syllables (left) and words (right) per prosodic phrase depending on Language, only in BI4 phrases and with no disfluencies (BI4-d). ^***^p < 0.001.

When only phrases ending in the BI4-type break and containing no disfluencies (BI4-d; see above) are considered, the difference between Czech and English phrases becomes even more pronounced [*χ*^2^(1) = 15.60, *p* < 0.0001]. As shown in the left part of Figure 2, prosodic phrases in Czech are, on average, by 1.72 (± 0.37 standard errors) syllables longer than those in English. Since the difference (as compared with the red confidence intervals in the left part of Figure 1) is attributable mostly to Czech, it seems that many of the shorter phrases ending in BI4 in Czech can be accounted for by some kind of disfluency (hesitation, prolongation, etc.). When expressed in words (in the right part of Figure 2), the tendency for longer phrases in English than in Czech is not significant [*χ*^2^(1) = 1.66, *p* > 0.1].

**Figure 3:**
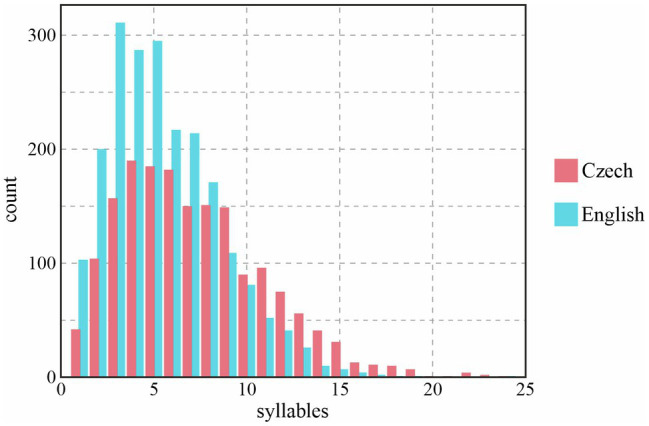
Distribution of number of syllables per prosodic phrase depending on Language, only in BI4 phrases and with no disfluencies (BI4-d).

Another perspective on the length of prosodic phrases in Czech and English is provided by the histogram in Figure 3; again, only BI4-d phrases are shown. We can see that phrases in both languages are most typically quite short, with the most frequent phrase length around four to five syllables. The number of phrases which extend beyond eight syllables declines steadily in both languages. However, beyond the ten-syllable mark, the counts are consistently higher for Czech than for English; in other words, longer phrases are much more likely to be found in Czech than English, and the difference between the two languages in the previous two figures is, at least partially, caused by the prevalence of such extra-long phrases in Czech.

**Figure 4:**
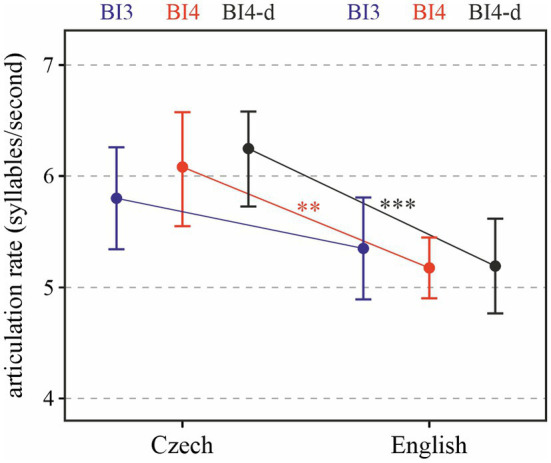
Articulation rate depending on Language and Prosodic break type (BI3 in blue, BI4 in red, and BI4-d in black). ^**^p < 0.05; ^***^p < 0.001.

Turning to articulation rate, we can see in Figure 4 that the Czech speakers were slightly faster in their delivery than the English ones. When we consider all data (i.e., the relationships shown in red and blue), the language factor significantly affected articulation rate [*χ*^2^(1) = 5.38, *p* < 0.05], with Czech being faster by 0.76 (± 0.29 standard errors) syllables per second on average.

The effect of prosodic break type is not significant [*χ*^2^(1) = 0.26, *p* > 0.6]. When only BI4-d phrases (i.e., those featuring no disfluencies) are taken into account, the difference is significant [*χ*^2^(1) = 8.69, *p* < 0.001], with the difference between the two languages approaching one syllable per second: on average 0.96 (± 0.31 standard errors) syllables/s.

### Melodic Patterning

**Figure 5:**
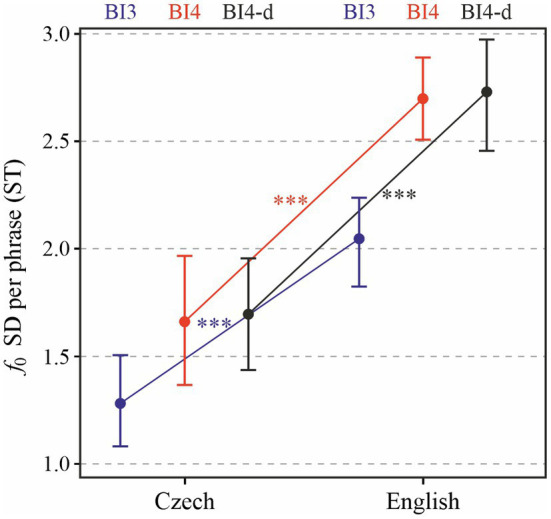
Standard deviation of *f*_0_ in each prosodic phrase depending on Language and Prosodic break type (BI3 in blue, BI4 in red, and BI4-d in black). ^***^p < 0.001.

First of all, we will examine melodic range in entire prosodic phrases. When expressed as the standard deviation of *f*_0_ in each prosodic phrase, Figure 5 shows that the speakers’ language affected pitch range significantly [*χ*^2^(1) = 15.98, *p* < 0.0001]. On average, *f*_0_ standard deviation in English was by 0.78 (± 0.15 standard errors) ST larger than in Czech. prosodic break type (BI4 or BI3) also significantly affected the standard deviation of *f*_0_ in prosodic phrases [*χ*^2^(1) = 26.74, *p* < 0.0001], with differences between speakers ranging between 0.08 and 0.94 ST. Post-hoc tests, whose results are indicated using asterisks in the figure, indicate significant differences between *f*_0_ standard deviation across languages: melodic range is higher in English than in Czech phrases ending in BI4 (*p* < 0.0001), as well as BI3 (*p* < 0.001). In addition, *f*_0_ standard deviation in minor (BI3) phrases is, in each language, significantly lower than in major (BI4) phrases (*p* < 0.0001).

The results differ only very little when BI4-d phrases are considered (i.e., those BI4-type phrases which feature no disfluencies, shown in black in Figure 5): language again turned out to be a significant predictor of *f*_0_ standard deviation [*χ*^2^(1) = 19.48, *p* < 0.0001], with mean values higher in English by 1.02 (± 0.19 standard errors) ST.

**Figure 6:**
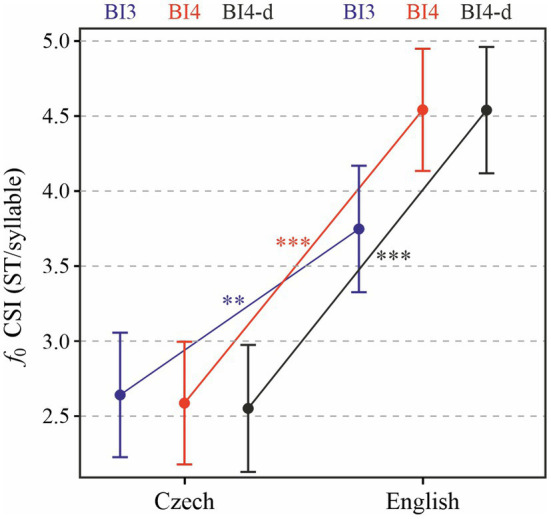
Cumulative slope index (CSI) of *f*_0_ in each prosodic phrase depending on Language and Prosodic break type (BI3 in blue, BI4 in red, and BI4-d in black). ^**^p < 0.05; ^***^p < 0.001.

In Figure 6, we provide a slightly different perspective on the melodic behavior of our TED speakers’ recordings. As explained in “Analyses”, the Cumulative Slope Index (CSI) does not reflect only the range of *f*_0_ values in a prosodic phrase, but rather captures potential multiple melodic movements within phrases, and may thus be a superior indicator of melodic variability. Overall, the effect of language on CSI is significant and similar in its magnitude to the standard deviation of *f*_0_ reported above [*χ*^2^(1) = 15.73, *p* < 0.0001]. On average, melodic variability as expressed by CSI is by 1.62 ST/syllable (± 0.27 standard errors) higher in English than in Czech. The analysis of the effect of prosodic break type yielded a singular fit and could not be carried out. The interaction between language and prosodic break type (BI4 and BI3) turned out to be significant [*χ*^2^(1) = 9.35, *p* < 0.01]. As for phrases ending in a BI4 break and containing no disfluencies, language significantly affected CSI values [*χ*^2^(1) = 24.29, *p* < 0.0001], with mean CSI higher by 1.99 (± 0.30 standard errors) ST/syllable in English than in Czech. Tukey post-hoc test results reveal that CSI values do not significantly differ between major (BI4) and minor (BI3) phrases in Czech, while in English CSI is significantly lower in minor than in major phrases (*p* < 0.0001). The main difference in melodic variability between the two languages therefore consists in the major prosodic phrases, regardless of the presence or absence of disfluencies (*p* < 0.0001).

**Figure 7:**
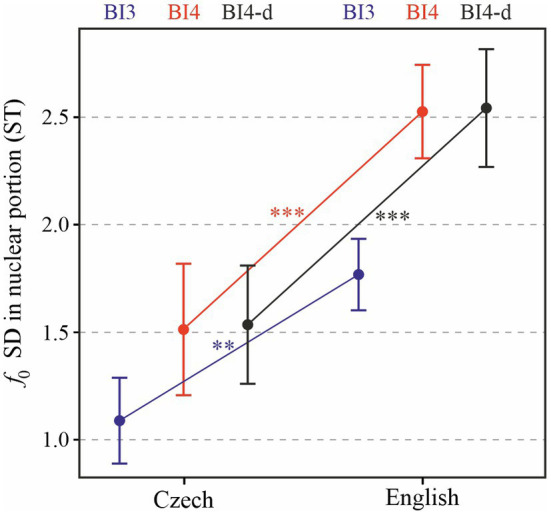
Standard deviation of *f*_0_ in the nuclear portion of each prosodic phrase, depending on Language and Prosodic break type (BI3 in blue, BI4 in red, and BI4-d in black). ^**^p < 0.05; ^***^p < 0.001.

Finally, as we mentioned at the end of “Research Questions”, it may be illustrative to consider melodic variability separately in the nuclear and pre-nuclear field, because the divergent impressions of English and Czech melodic patterning may be more strongly related to one or the other, as compared with the entire prosodic phrase. The results of standard deviation of *f*_0_ in the nuclear portion of prosodic phrases are shown in Figure 7. The effect of language is significant [*χ*^2^(1) = 15.97, *p* < 0.0001], with *f*_0_ standard deviation higher by 0.67 (± 0.13 standard errors) ST on average. The effect of prosodic break type also turned out to be significant [*χ*^2^(1) = 27.99, *p* < 0.0001]. Tukey post-hoc tests show that the difference between BI3 and BI4 is significant both in Czech and in English (*p* < 0.0001) and that cross-language comparisons by prosodic break type are also significant (*p* < 0.0001 for BI4 and *p* < 0.01 for BI3). When only BI4-d phrases are considered, the results for *f*_0_ standard deviation in the nuclear portion are similar [*χ*^2^(1) = 17.83, *p* < 0.0001], with mean values being higher by 1 (± 0.2 standard errors) ST.

**Figure 8:**
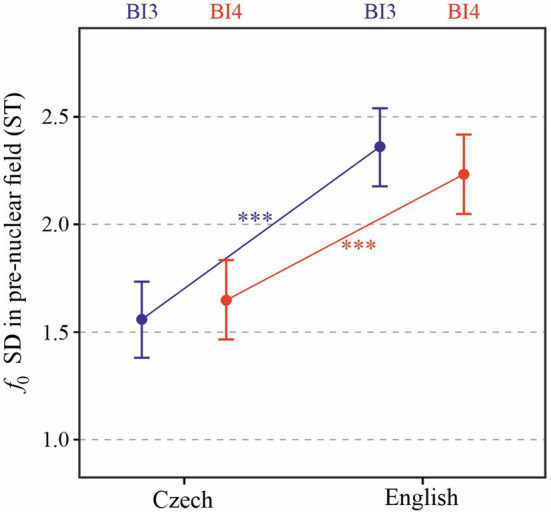
Standard deviation of *f*_0_ in the pre-nuclear field of each prosodic phrase depending on Language and Prosodic break type (BI3 in blue, BI4 in red, and BI4-d in black). ^***^p < 0.001.

Results for standard deviation of *f*_0_ in the pre-nuclear field of prosodic phrases are shown in Figure 8. The effect of language is significant [*χ*^2^(1) = 18.03, *p* < 0.0001], with *f*_0_ standard deviation higher by 0.7 (± 0.12 standard errors) ST on average. Unlike in previous analyses, the effect of prosodic break type is not significant [*χ*^2^(1) = 0.08, *p* > 0.5]; however, the interaction between the two factors does reach significance [*χ*^2^(1) = 4.78, *p* < 0.05]. As indicated using the asterisks in the figure, post-hoc comparisons confirm significant differences between *f*_0_ standard deviation in Czech and English for both BI3-type and BI4-type phrases (*p* < 0.0001).

### Individual Prosodic Profiles

**Figure 9:**
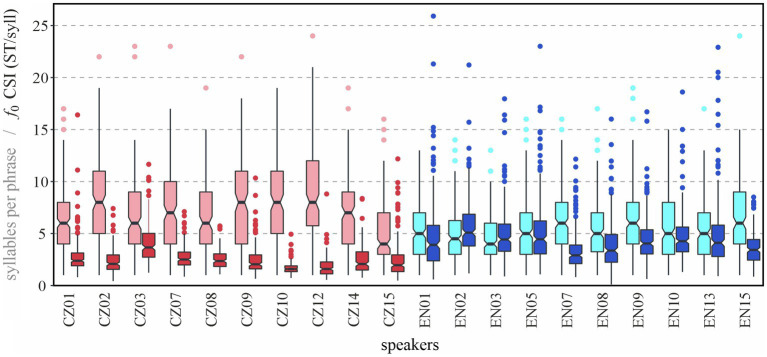
Prosodic phrase length in syllables (boxplots on the left in lighter colors) and cumulative slope index (CSI) of *f*_0_ (boxplots on the right in darker colors) for individual speakers in BI4-d phrases.

The aim of this section is to provide a glimpse at individual variability between our speakers by placing length of prosodic phrases and melodic variability (CSI) next to each other. The comparison is shown using boxplots in Figure 9 (Note, first, that only BI4-d phrases are shown and second, that the scales of the two variables are identical when outliers are included).

The average tendencies reported in the previous sections—longer phrase durations (always on the left for each speaker) and lower melodic variability (on the right) in Czech than in English—are clearly visible from the plot. It is not surprising that the mean values conceal considerable individual variability. In terms of phrase length, speaker CZ15’s distribution is quite similar to that of most English speakers. On the other hand, phrases of speakers like CZ02 or CZ12 are on average twice as long as those of speaker CZ15. Turning to melodic variability, we can see that speakers EN07 and EN15 differ very little from some of the Czech speakers. In Czech, speaker CZ10’s distribution is extremely narrow, with all prosodic phrases manifesting a CSI of *f*_0_ lower than 5 ST/syllable.

It is also interesting to consider the highest values of both variables. For phrase length, while all phrases longer than 15 syllables correspond to outliers in English (represented by individual dots in Figure 9), and there are only nine of such phrases, one half of the Czech speakers’ “normal” values (i.e., those within the boxplot whiskers) exceed 15 syllables, and there are 50 phrases in the Czech material exceeding 15 syllables in length. For melodic variability, it is obvious at first sight that the English material features more outliers, and their values reach higher values.

## Discussion

This study has focused on prosodic patterning in good public speakers active on the TED Talk platform, with the prosodic phrase as the domain of analysis. We analyzed ten speakers of Czech and ten of American English, two languages which differ considerably in their prosodic patterning. Our objective was to find whether the language-specific differences persist also in this genre—public speaking in an entertaining manner in front of an audience—or whether genre-specific requirements will bring speakers of the languages closer to each other in terms of their temporal and melodic behavior.

Our results indicate differences between Czech and English public speakers in both the temporal and melodic domain. As for the former, the length of prosodic phrases, when expressed in the number of syllables, differs especially in major prosodic phrases (those followed by a BI4 break). However, the phrases were considerably shorter in both languages than those reported by Volín (2019) for news reading on the radio, as shown by the comparison in Table 1. The prevalence of rather short phrases was clear in the histogram in Figure 3, and it is clear that the genre exerts a strong effect on the prosodic segmentation of speech: good speakers in both Czech and English use shorter prosodic phrases, they divide their flow of speech more often by prosodic breaks to achieve a better effect on the audience. Some studies also indicate that shorter prosodic phrases may be characteristic of skilled public speakers (Strangert, 2005). This is not surprising, as a more structured speech makes the process of understanding the message easier, and shorter segments are easier to process. Short phrases often appeared in contexts when our public speakers used emphasis; they frequently realized such words in separate prosodic phrases, as shown by the following examples from our data:

1. It turned out to be | shame.
2. A říká se tomu | exotika (*and it’s called | exotica*).

**Table 1:** Mean length of prosodic phrases (in syllables) in English and Czech in news reading (Volín, 2019) and in TED speakers (this study; only major phrases with no disfluencies are included).

	English	Czech
Radio newsreaders	7.8	10.8
TED speakers	5.5	7.2

Let us consider the question of language- vs. genre-specific tendencies mentioned above. As shown in Table 1, phrases are longer in Czech than in English by 31% in our material, while in Volín’s data, this difference amounts to 38%. It appears, therefore, that phrasing is influenced by both genre (resulting in shorter phrases in public speaking than in news reading) and language (with phrases longer in Czech than in English).

As for melodic patterning, the results of this study point to lower values of pitch range (standard deviation of *f*_0_) and overall melodic variability (cumulative slope index, CSI) in Czech than in English, thus lending support to previous studies (especially Volín et al., 2015), as well as informal observations. To make our results comparable with those of Volín et al. (2015), we also calculated the 80-percentile ranges of the *f*_0_ data; the comparison is shown in Table 2. It can be seen that pitch range is narrower in our data when compared with news reading, although the difference is quite small: it amounts to a little over one quartertone in English and slightly exceeds one semitone in Czech. In melodic patterning, our data therefore suggest that language-specific tendencies exert a greater influence on the speakers’ pitch range than the genre.

**Table 2:** 80-percentile range of *f*_0_ (in semitones) in English and Czech in news reading (Volín et al., 2015; male and female values are averaged here) and in TED speakers (this study; only major phrases with no disfluencies are included).

	English	Czech
Radio newsreaders	7.60	5.65
TED speakers	7.05	4.48

Unfortunately, it is not possible to compare our results with studies reported in “Charismatic Speech and Prosody” which targeted pitch range: none of these report absolute values, only correlation coefficients relating pitch range to perceived charisma (or another measure like the number of subscriptions).

In this study, we adopted a closer look at melodic variation within a prosodic phrase by considering not only the entire phrase as a unit, but also dividing it into the nuclear part (carrying the nuclear tone) and the pre-nuclear field. Specifically, we hypothesized that the impression of flat melody in Czech may be due to little variation in the pre-nuclear field, especially when combined with the tendency to produce longer prosodic phrases in Czech. However, our results do not suggest a difference in the standard deviation of *f*_0_ in the pre-nuclear or nuclear portion of prosodic phrases (see Figures 7, 8): in both Czech and English, *f*_0_ standard deviation appears to be quite similar in both portions of prosodic phrases. In fact, it seems to be English where melodic range is smaller in the pre-nuclear field than in the nuclear part. It would be interesting, however, to conduct comparable analyses on spontaneous dialogs or other speaking styles in Czech and English: the melodic difference between the pre-nuclear and nuclear field may become more pronounced there.

There are some minor findings which are worth commenting on. First of all, it should be noted that the length of prosodic phrases does not significantly differ between the two examined languages when it is expressed in words. The same result was reported by Volín (2019), as already mentioned in “Prosodic Patterns in English and Czech”. This seems to be due to the different morphological type of Czech and English, with Czech using inflections where English uses individual words.

In the current study, we analyzed separately phrases ending in a weaker, BI3-type of break (also referred to as minor phrases above) and those ending in a stronger, BI4-type of break (major phrases). Our results showed a significant effect of prosodic break type, with minor phrases being shorter and having a narrower pitch range than major phrases in both languages. In addition, we identified those phrases which included a disfluency (most frequently these were minor phrases); subsequently, we presented results for phrases without any disfluencies (marked BI4-d). One of the reasons for this was our expectation that these “full-fledged” phrases are what is typically analyzed in most other studies; that is, why the above comparisons with studies by Volín and colleagues featured only these BI4-d phrases.

Finally, articulation rate turned out to differ between our Czech and American English speakers; interestingly, the mean values correspond quite closely to those reported in literature, even for other speaking styles or genres. The mean articulation rate (in BI4-d phrases only) in our Czech data is 6.2 syllables per second, similar to the 6.1 syll/s reported by Veroňková and Poukarová (2017) for Czech newsreaders. Our American English speakers’ mean articulation rate was 5.2 syll/s, while that in the read speech examined by Baese-Berk and Morrill (2015) was 4.9 syll/s. Note that while these are relatively fast speech rates, good quality speaking has been associated with faster speaking (see “Charismatic Speech and Prosody”).

The comparison of individual tendencies in “Individual Prosodic Profiles” provided a useful perspective on our data, and it is crucial for the concept of a good or charismatic speaker. All our speakers were chosen by listeners from a larger dataset as high-quality speakers (*cf.* “Material” for speaker selection). To take but one example, given that speaker CZ10’s melody is flattest and his phrases belong among the longest, it seems obvious that being a “good speaker” involves a constellation of a number of factors which may partially compensate for each other. Based on our current knowledge (see “Charismatic Speech and Prosody”), temporal and melodic variability appear to be crucial but not the only components of such a constellation, and speaker charisma should be treated as a multidimensional phenomenon.

To conclude, the current study has confirmed previous studies and informal observations that the Czech language is prosodically more monotonous than English but extended them by analyzing skilled speakers who have been selected for their high-quality delivery. In our TED speakers, Czech prosodic phrases are approximately 30% longer than English ones and, at the same time, variation in the melodic domain is much lower, with *f*_0_ standard deviation smaller by one semitone and melodic variability (expressed by CSI) by nearly two semitones smaller in Czech than English. It is not surprising, then, that such a combination results in the perception of monotonousness or flatness; naturally, this will be particularly salient to listeners whose native language manifests greater melodic variability. There seems to be a smaller difference between the two languages in phrase length than in other genres, showing a combined effect of language and genre, but pitch range remained mostly language-specific. The natural follow-up to this study would be moving from skilled, charismatic speakers to ordinary ones and their prosodic behavior in everyday conversations.

The implications of our study extend to speakers who strive for high-quality performance. It is clear that prosodic rendition of one’s speech—especially the length of prosodic phrases, melodic variability, and speech rate—is a vital aspect of speech performance. Given the fact that these prosodic aspects of speech are, to a considerable extent, language-specific, speakers should take special care when they deliver a speech or give a presentation in a foreign language: what may sound like charismatic speaking in one language may sound flat and disinterested or, on the other hand, affected and insincere in another.

## Data Availability Statement

The raw data supporting the conclusions of this article will be made available by the authors, without undue reservation.

## Author Contributions

All authors listed have made a substantial, direct, and intellectual contribution to the work and approved it for publication.

## Funding

This study was supported by the Czech Science Foundation project “Prosodic Phrase in Current Spoken Czech: Meaning, Balance, Stochastic Patterns” (no. GA 21-14758S) and from the European Regional Development Fund-Project “Creativity and Adaptability as Conditions of the Success of Europe in an Interrelated World” (no. CZ.02.1.01/0.0/0.0/16_019/0000734).

## Conflict of Interest

The authors declare that the research was conducted in the absence of any commercial or financial relationships that could be construed as a potential conflict of interest.

The handling editor declared a past co-authorship with one of the authors RS.

## Publisher’s Note

All claims expressed in this article are solely those of the authors and do not necessarily represent those of their affiliated organizations, or those of the publisher, the editors and the reviewers. Any product that may be evaluated in this article, or claim that may be made by its manufacturer, is not guaranteed or endorsed by the publisher.

## Footnotes

^1^This is not to suggest, however, that speakers on the TED platform do not differ in their speaking quality and charismatic impact, as confirmed, among others, by MacKrill et al. (2021).
